# Supplementary material for: Theory of minds: managing mental state inferences in working memory is associated with the dorsomedial subsystem of the default network and social integration
Source: Soc Cogn Affect Neurosci. 2020 Feb 12;15(1):63–73. doi: 10.1093/scan/nsaa022 (PMC7171370; doi:10.1093/scan/nsaa022)
Supplement: scan-19-205-File009_nsaa022 [file scan-19-205-file009_nsaa022.docx]

**Supplementary Materials**

**
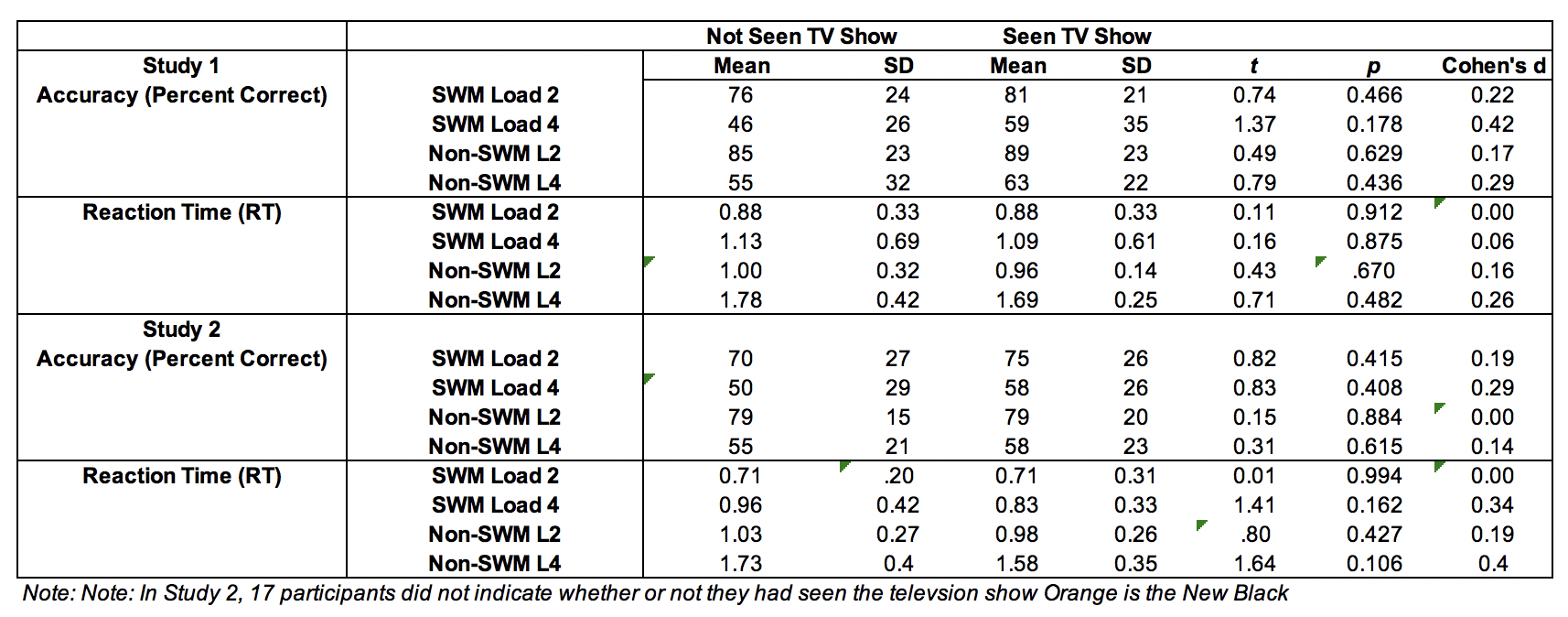
**

*Supplementary Table 1. Task performance in Studies 1 and 2 for participants who had and had not previously seen the television show Orange is the New Black.*

*
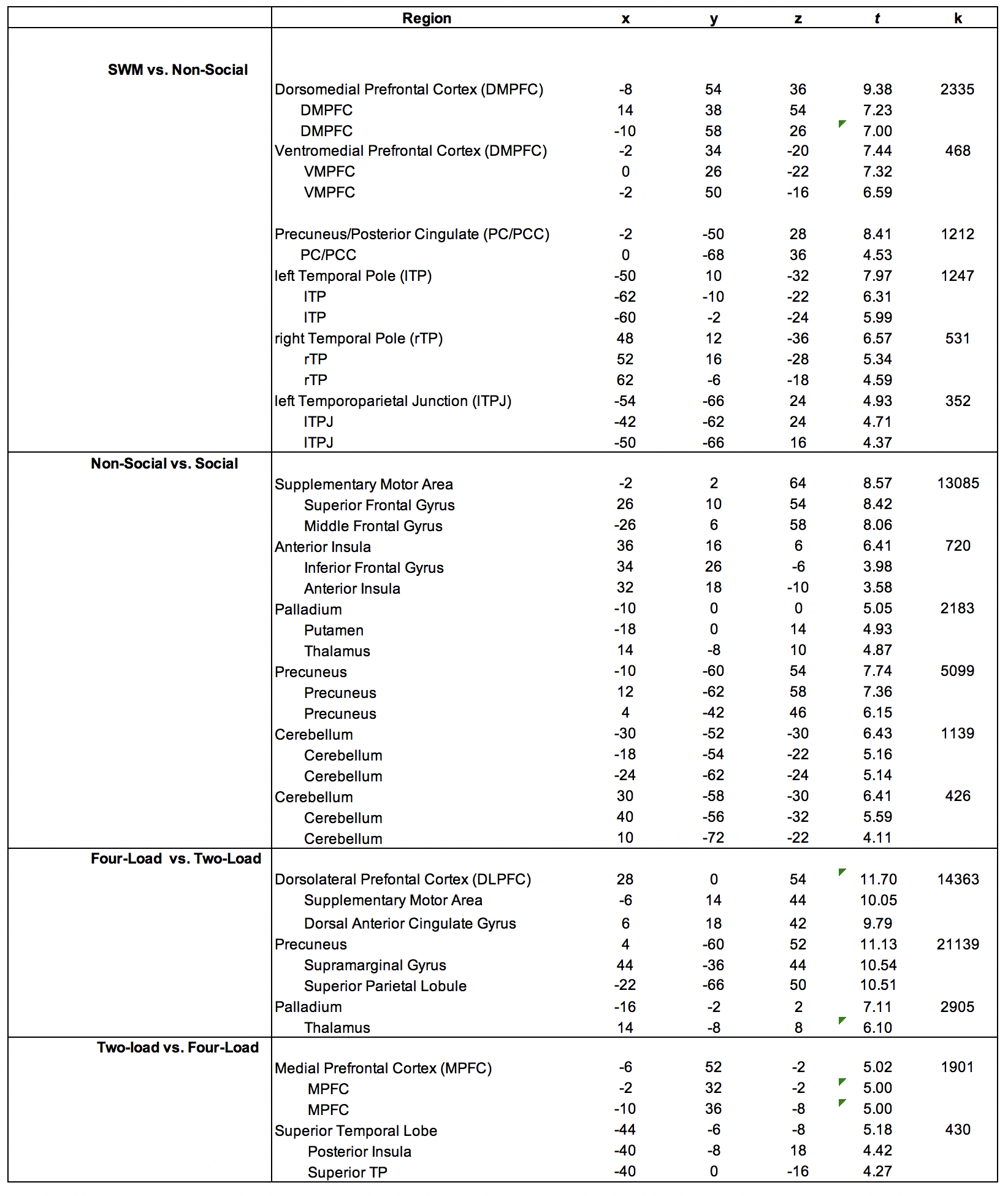
*

*Supplementary Table 2. Clusters of neural activity associated with Social vs. Non-Social working memory trials and Four-load vs. Two-Load working memory trials.*

**
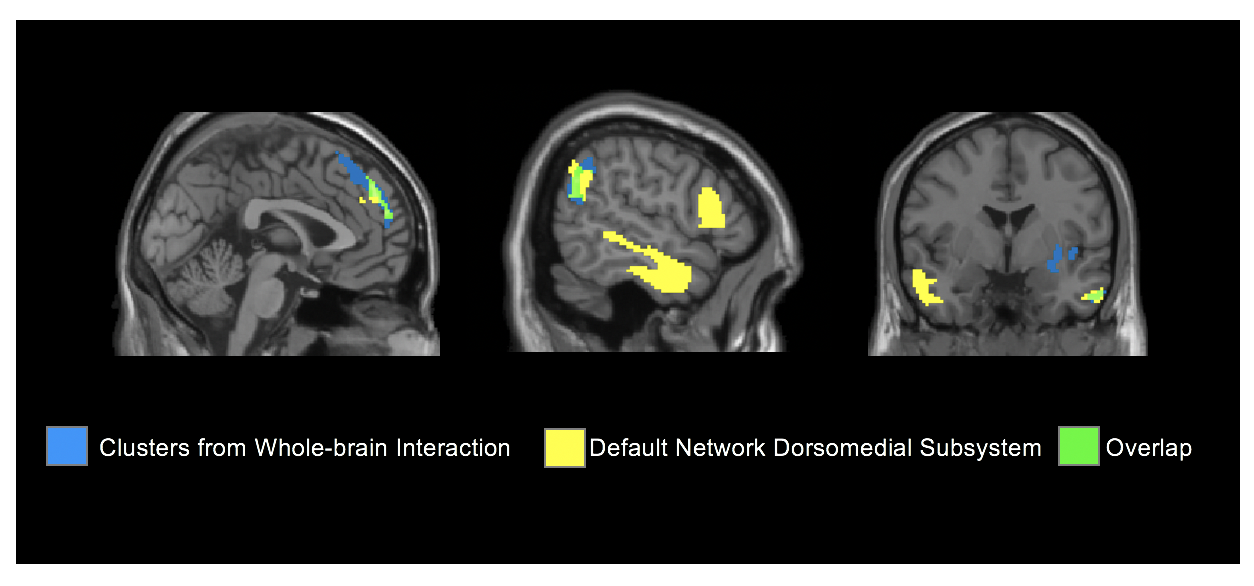
**

*Supplementary Figure 1. Clusters of neural activity from the whole-brain interaction comparing greater activity in response to SWM four-load (vs. two-load) relative to non-SWM four-load (vs. two-load) trials (in blue), the dorsomedial subsystem of the default network defined by Yeo et al. (2011; yellow) and their overlap (green). There is overlap in dorsomedial prefrontal cortex, tempoparietal junction, and temporal poles.*

**
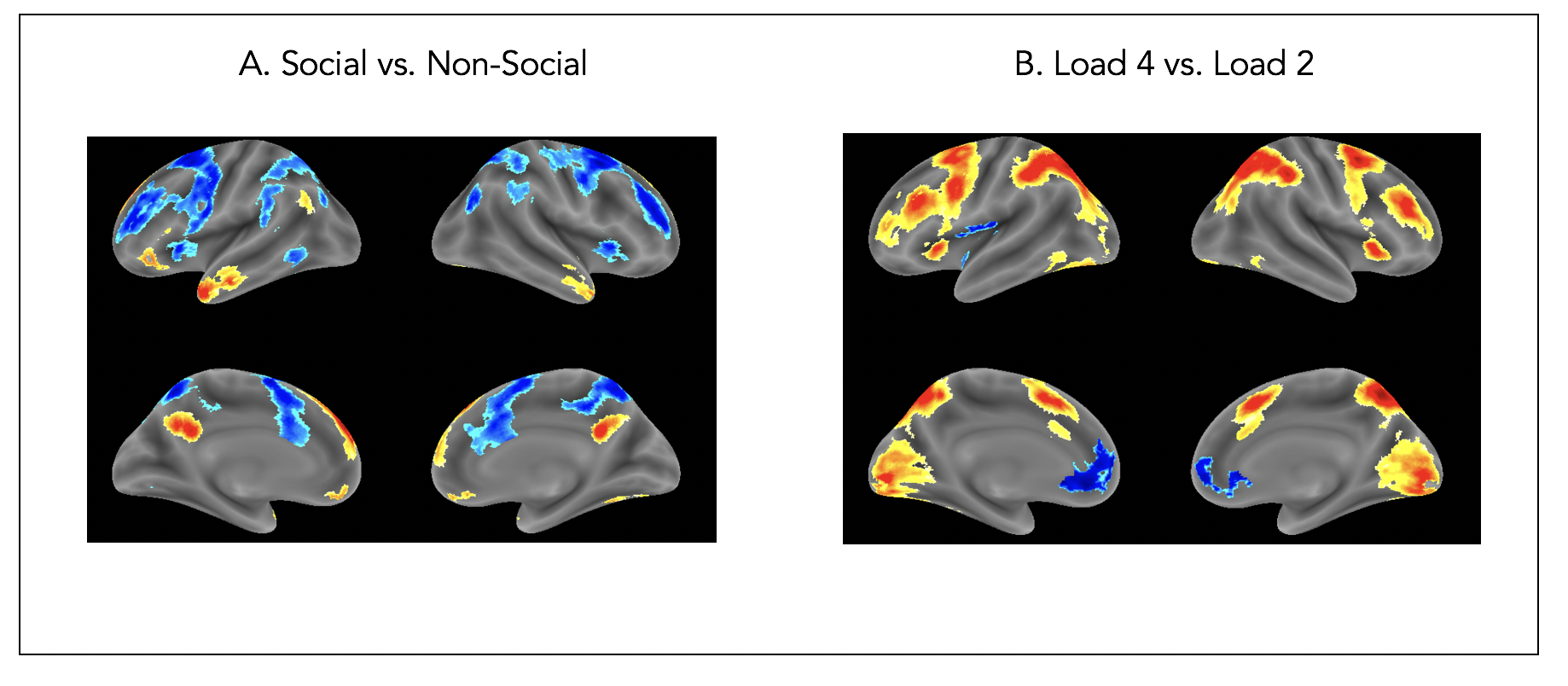
**

*Supplementary Figure 2. Main effect analyses. Warm colors indicate activation increases and cool colors indicate activation decreases. Panel A shows neural activity associated with SWM vs. non-SWM trials. Panel B shows neural activity associated with four-load vs. two-load trials.*

**
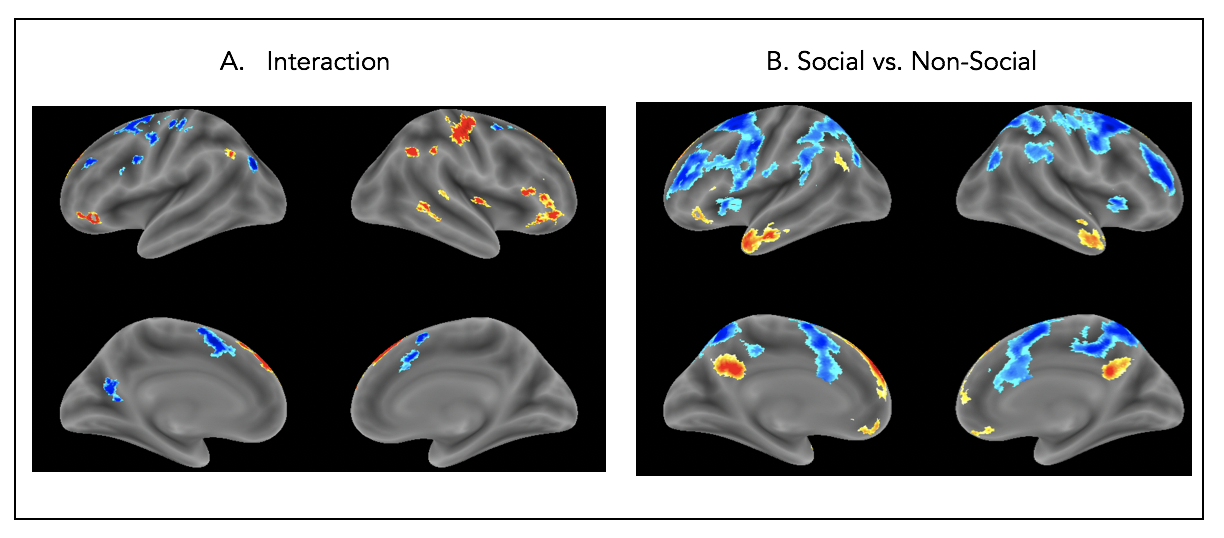
**

*Supplementary Figure 3. Contrasts in which SWM and non-SWM trials showed different patterns of neural activity, controlling for reaction time (RT). Warm colors indicate activation increases and cool colors indicate activation decreases. Panel A shows results for the interaction contrast testing for neural activity more strongly associated with SWM four-load (vs. two-load) relative to non-SWM four-load (vs. two-load) trials. Panel B shows results for the contrast comparing SWM relative to non-SWM trials, collapsed across load level.*

**
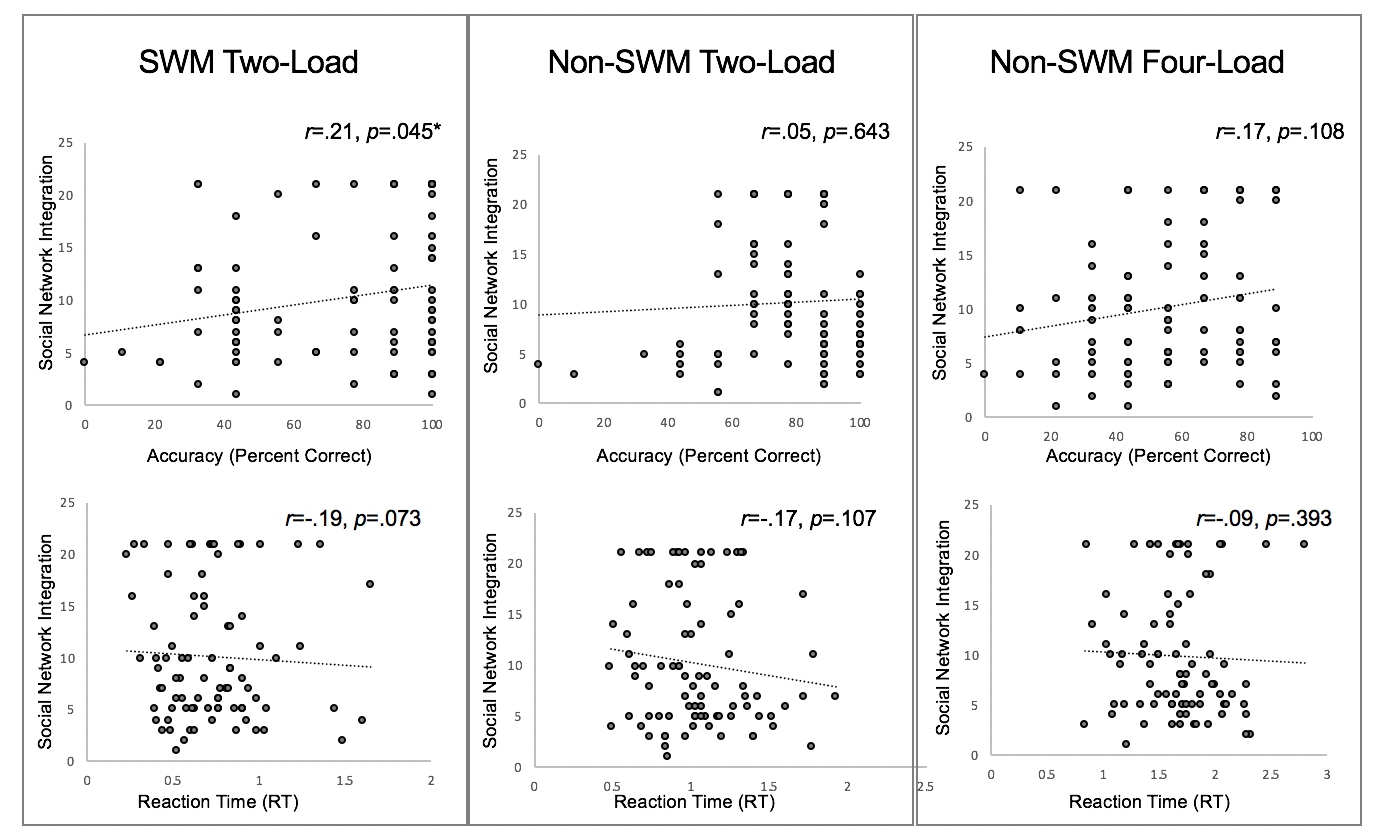
**

*Supplementary Figure 4. Scatter plots showing the relationship between social network integration (y-axis) and* *SWM Two-Load, non-SWM Two-Load and non-SWM Four-Load trials.*
